# Intermittent water supply and self-rated health in rural China's karst region

**DOI:** 10.3389/fpubh.2023.1054730

**Published:** 2023-03-02

**Authors:** Guoyong Wu, Jianwei Cheng, Fan Yang, Noman Riaz

**Affiliations:** ^1^China Center of Western Capacity Development Research, School of Economics, Guizhou University, Guiyang, Guizhou, China; ^2^Guizhou Grassroots Social Governance Innovation High-End Think Tank, Ecological Civilization (Guizhou) Research Institute, Guiyang, Guizhou, China; ^3^Rural Revitalization Institute in Karst Region of China, Guizhou University, Guiyang, Guizhou, China

**Keywords:** intermittent water supply, self-rated health, water storage behavior, sanitary water habit, China

## Abstract

**Introduction:**

With rapid economic and social development, surging demand for domestic water, and the increasing shortage of water resources, the applications of intermittent water supply systems have become more common in rural China and other developing countries. The accompanying hygiene risks require our more attention.

**Methods:**

Based on the Grossman model, this paper conducted an IV-Oprobit model to investigate whether and how intermittent water supply affect rural residents' self-rated health status. Our data came from “China Karst Rural Economic Survey (CKRS)”, which covers 8 provinces and 641 villages in rural China's karst region.

**Results and discussion:**

We found that: (1) Intermittent water supply has adverse effects on the self-rated health status of rural residents. Compared with the rural residents under continuous water supply, the probability of “fair” health status under intermittent water supply significantly increases by 18.2%, while the probability of “excellent” significantly reduces by 58.8%. (2) Residents' water storage behavior and sanitary water habit are important mechanisms for intermittent water supply to affect residents' self-rated health status; (3) Intermittent water supply has a greater impact on the self-rated health of females and the groups with lower education levels. The results of our study have the following policy implications: relevant departments should make a rational plan about water supply methods and improve related supporting measures; we should strengthen health education for rural residents on water behavior to standardize their water storage and sanitary water behavior; government should enhance the pertinence of policy implementation and favor specific measures to specific populations.

## 1. Introduction

Water is the basic source of life, and health is the most important indicator of a happy life. The rural drinking water safety project is a basic project to ensure the water quality for rural China residents. The project can achieve social and economic benefits and also promote the quality of life for rural residents (1). Due to frequently changing extreme weather conditions and rapid urbanization, the demand for domestic water for rural residents is also increasing. The pressure on the supply of water resources has further increased (2, 3).

The intermittent water supply, which refers to the water is provided for only limited durations and does not meet the requirements of supplying water to users 24/7. The intermittent water supply has been applied in more and more regions all over the world, especially in rural areas of developing countries, due to its remarkably flexible effect on the control of water supply (4, 5). According to statistics, intermittent water supply is serving more than 300 million residents worldwide (3). Evidence suggests that intermittent water supply significantly poses potential health risks. Most studies focused on water quality, including microbiological, chemical, physical, and aesthetic properties (6–8). When concerning individual's water behavior, there were some disagreements: Some scholars believed that an effective water supply time limit can reduce waste, promote water storage behavior, and reduce the households' average daily water consumption. Furthermore, it decreased the health risks to a certain extent by promoting households to develop the adaptive behavior (9). However, some scholars found that the limitation of water supply time can promote residents' sanitary water behavior, including the use of alternative water sources, storage water, and reducing sanitary water, all of which would increase the potential risks of water safety (10, 11). Meanwhile, intermittent water supply has always been considered one of the main pollution sources in China's water supply network (12). Whether and how intermittent water supply affects residents' self-rated health still needs further analysis.

As evident from the published literature in this field, intermittent water supply and individual water behavior have not been comprehensively applied to the study of rural residents' health through empirical analysis, especially in China, the largest developing country in the world. Relevant studies in China mostly focused on water safety (13, 14), water quality (15), water supply sanitation (16), etc. In terms of the research content, there are seldom studies quantifying the intermittent water supply's actual effect on self-rated health. Some existing studies only discussed the health risks in household water activities (17). Or from physicochemical perspectives to discuss the health risks of intermittent water supply concerning specific diseases, which did not take individual's behavior into consideration (18–20). In research methods, there is a lack of a health demand model for water users to quantify the effect of water supply and other factors on self-rated health. Most of the studies tended to assess health risks by examining water quality's physical and chemical properties through experiments. And some studies treated sanitary water behavior as one of the factors that may affect self-rated health, which did not further discuss the effects (21). As for the research sample, most of previous study samples only covered specific counties or regions (5, 10, 17, 20). So, it is easy to cause disagreements mentioned above due to geographical differences, unique topographical conditions, and other subjective reasons.

The main objective of this paper is to quantify the effect of intermittent water supply on self-rated health and discuss its possible mechanism by studying residents' water behavior. Our paper contributes to the growing body of literature on the study of water supply and public health. Based on the Grossman model, we adopt a health demand model of residents to quantify the effect of intermittent water supply on self-rated health. Moreover, we investigate the mechanisms underlying the effects and examine that the residents' water storage behaviors and sanitary water habits are the main channels, which could also provide new perspective in the further study of water behavior. We use observational data from the “China Karst Rural Economic Survey (CKRS)”, which covers 81 counties/districts, 641 villages, and 8 provinces in the karst region of China. Due to the distinctive landforms, the uneven spatial and temporal distribution of karst water resources, and poor surface water holding capacity, there are underutilization of water resources and typical “engineering water shortage” problems in the karst areas (22, 23), which increases the difficulties of water supply. Therefore, our study in the karst region will be highly representative and valuable for water supply management and public health policy-making in rural China and other developing countries.

The remainder of the paper is organized as follows. Section Materials and methods describes the theoretical framework of this study, outlines our materials and estimation strategy. Section Results presents our empirical findings, and Section Discussion presents further discussions about the effects in different groups. Section Conclusion concludes and discusses the policy implications of our findings.

## 2. Materials and methods

### 2.1. Theoretical foundations and hypotheses

Health in the framework of economics has been studied with a variety of semantics. Welfare economics believes that health is a basic human right (24), Amartyason's development theory believes that health belongs to basic freedom (25), and human capital theory believes that health is a basic human capital. Since Grossman constructed the health demand model for the first time (26), scholars have carried out research on health problems based on this theoretical basis (27). In the Grossman model, health is regarded as a durable capital good. Everyone is born with a certain health capital stock. This capital stock will be depreciated with the change of relevant factors (such as individual age, etc.), and can also increase by making health investment (such as an increase in health services, etc.). Suppose a consumer's utility function at various periods in his life is:


(1) $$\begin{array}{l} U=U\;\left(\varphi_{t}H_{t},{\;Z}_{t}\right),t=0,1,\ldots ,n \end{array}$$

Where *H*_*t*_ represents the health capital stock of consumers in the *t* period, φ_*t*_ represents the income per unit of health capital, and φ_*t*_*H*_*t*_ represents the health capital consumed in the *t* period, which is the total service value provided by *H*_*t*_. And represents the quantity of all the other goods consumed by the consumer in period t. Health as a durable capital good, its capital increment is:


(2) $$\begin{array}{l} H_{t+1}-H_{t}=I_{t}-\delta_{t}H_{t} \end{array}$$

Where *I*_*t*_ is the health investment in period *t*, δ_*t*_ is the depreciation rate of healthy capital in period *t* (0 < δ_*t*_ < 1), assuming it is an exogenous variable but varies with the age of the consumer. Health investment *I*_*t*_ and consumption of other goods *Z*_*t*_ are jointly determined by the following household production functions:


(3) $$\begin{array}{l} I_{t}=I_{t}\;\left(M_{t},\;{\;TH}_{t};{\;E}_{t}\right) \end{array}$$

(4) $$\begin{array}{l} Z_{t}=Z_{t}\;\left(Y_{t},\;{\;T}_{t};{\;E}_{t}\right) \end{array}$$

Among them, *M*_*t*_ and *Y*_*t*_ represent a series of goods or services that consumers can buy, which can be used as inputs to produce *I*_*t*_ and *Z*_*t*_. *TH*_*t*_ and *T*_*t*_ represent the time input to produce *I*_*t*_ and *Z*_*t*_. *E*_*t*_ denotes the other human capital except for health. At the same time, consumers will also face certain budget constraints and time constraints. The constraint equations are shown in equations (5) and (6) respectively:


(5) $$\begin{array}{l} \overset{n}{\underset{t=0}{\sum }}\frac{P_{t}M_{t}+Q_{t}Y_{t}}{{\left(1+r\right)}^{t}}=\overset{n}{\underset{t=0}{\sum }}\frac{W_{t}TW_{t}}{{\left(1+r\right)}^{t}}+W_{0} \end{array}$$

(6) $$\begin{array}{l} TW_{t}+TL_{t}+TH_{t}+T_{t}=\partial \end{array}$$

Where *P*_*t*_ and *Q*_*t*_ denote the price of *M*_*t*_ and *Y*_*t*_ respectively. *W*_*t*_ denotes the wage rate. *TW*_*t*_ denotes the number of working hours, and *W*_0_ denotes the initial accumulation of wealth. ∂ denotes the time consumers possess in different periods, and *TL*_*t*_ denotes the time lost due to health reasons. Therefore, the above formulas (1)–(6) construct the health demand model of consumers, and consumers need to maximize utility under a certain budget and time constraints.

On this basis, since the pure investment model has the characteristics of weak assumptions and strong predictions (18), this paper draws on the equilibrium conditions obtained by Zhong and Zhengang (19) based on the pure investment model:


(7) $$\begin{array}{l} \left[W_{t}+\left(U_{ht}/m\right){\left(1+r\right)}^{t}\right]G_{t}{\pi_{t-1}}^{-1}=r+\delta_{t} \end{array}$$

**Figure 1:**
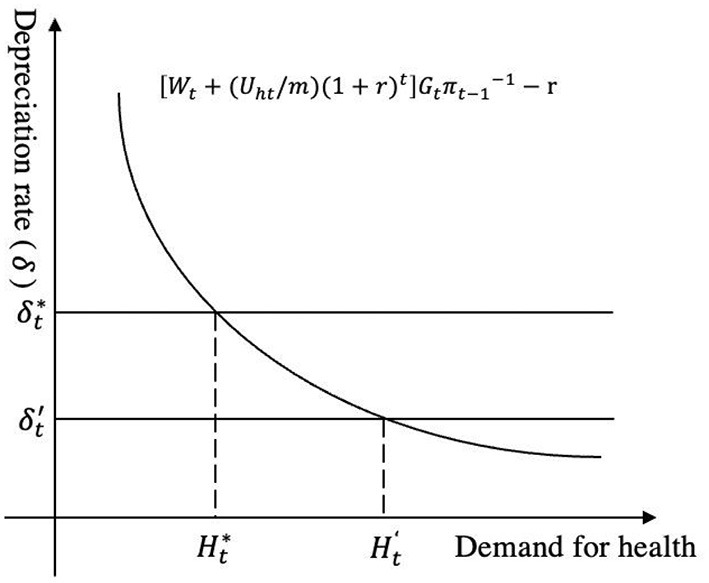
Equilibrium analysis of health demand.

Where *G*_*t*_ = ∂*TL*_*t*_/∂*H*_*t*_, represents the marginal product of health; *U*_*ht*_ = ∂*U*/∂*H*_*t*_, represents the marginal utility of health; *m* represents the marginal utility of money; π_*t*−1_ represents the shadow price of health. The left side of equation (7) represents the marginal benefit of health, which is composed of two parts: the direct utility of health $(U_{ht}/m){\left(1+r\right)}^{t}/\pi_{t-1}$ and the monetary benefit *W*_*t*_/ π_*t*−1_. The right side of the equation is composed of interest rate and depreciation, representing the marginal cost of health. Therefore, the optimal health demand $H_{t}^{{}^{\prime }}$ is determined by the intersection of the marginal revenue curve and marginal cost, as shown in the Figure 1.

Existing literature paid more attention to the change of depreciation rate δ_*t*_, and some studies found that factors such as pollution conditions and living conditions would affect the physical environment of health investment, resulting in an increased depreciation rate (28, 29). Similarly, our study believes that the intermittent water supply will affect the physical environment of rural residents. And it will undermine the physical environment of health investment through the issues of water quality, water quality assurance, sanitary water habits, etc. Then it will increase the depreciation rate. As is shown in the figure, the increase from the equilibrium state $\delta_{t}^{{}^{\prime }}$ to $\delta_{t}^{*}$, causes the marginal cost curve to move upwards. At $H_{t}^{*}$ where a new equilibrium is reached, and the demand for health decreases. Therefore, this study proposes:

**Hypothesis 1:** Intermittent water supply has adverse effects on the self-rated health status of rural residents.

**Figure 2:**
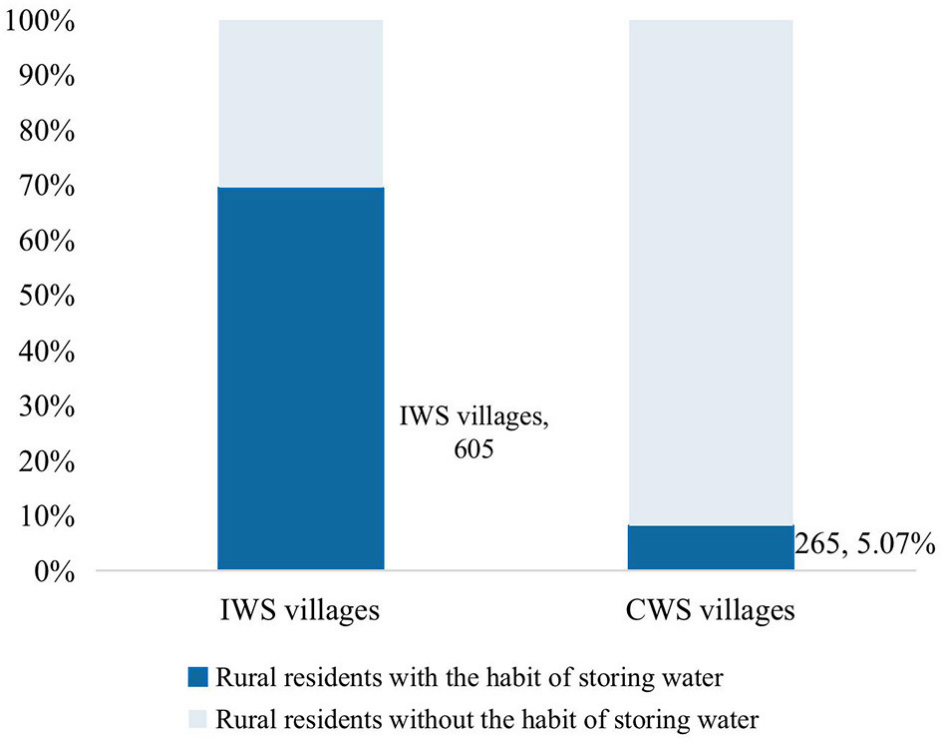
Water storage behavior under different water supply methods.

At the same time, the water supply method does not directly “produce” health. It is of great significance to discuss its probable mechanisms for formulating reasonable policies in the future. Through literature review and survey data analysis, we find that “water storage behavior” might be an important channel through which water supply affects residents' self-rated health. We mainly based on the following two aspects of consideration. Firstly, the proportion of rural residents with water storage behavior in intermittent water supply (IWS) areas has significantly increased compared to those in continuous water supply (CWS) areas (Figure 2). The correlation test also shows that intermittent water supply will significantly promote rural residents' water storage behavior. Secondly, previous studies have shown that water storage behavior can significantly increase hygiene risks from the aspects of the water storage environment, container, and duration. It has a certain impact on the health status of water users (18, 20). Therefore, this paper believes that there is mechanism of “intermittent water supply—water storage behavior—self-rated health status”. As a result, Hypothesis 2 is proposed:

**Hypothesis 2:** Intermittent water supply adversely affects health by promoting rural residents' water storage behavior.

Studies have shown that when the duration of water supply is limited, rural residents' water behavior, especially the sanitary water habit (handwashing, bathing, etc.), will also be affected to a certain extent (10, 11), thereby increasing the hygiene risks. In our survey, when we asked, “why don't you choose to take sanitary water behavior (drink boiled water, wash hands before and after meals, etc.)”, 45.5% of the respondent rural residents' answer was “inadequate water supply”, which also corroborated that “sanitary water habit” may be an important mechanism for the impact of water supply on health. However, the “sanitary water habit” is a behavior developed by the long-term daily accumulation with more complicated influencing factors and formation mechanisms. Therefore, our study believes that “sanitary water habit” is the mechanism of the impact of water supply on residents' health. Its role is not only reflected in the conduction mechanism but also has a certain regulatory effect. Therefore, we propose:

**Hypothesis 3**: Intermittent water supply will have a different impact on the self-rated health status of the residents with different sanitary water habits.

### 2.2. Data sources

Our data come from the “2020 China Karst Rural Economic Survey (CKRS)”. We conducted the survey through face-to-face interviews with households, covering 8 provinces and cities with widely distributed karst landforms. The data includes Chongqing City, Guangxi Zhuang Autonomous Region, Yunnan, Guizhou, Hubei, Hunan, Sichuan, and Guangdong Province, a total of 31 cities, 81 counties/districts, 164 towns/townships, and 641 villages. They contain rich information on villages and communities, allowing researchers to study rural social and economic development in the karst region.

To ensure the sample can reflect the overall situation of the research area as much as possible, we conducted this survey by using the PPS sampling method in the PUS sampling frame to select the first tier of samples (districts/counties), and a total of 40 counties were selected. The village houses in the counties had been selected by sorting number, and no < 4 natural villages had been selected in each county. The PPS method had used to randomly sample the village houses, and finally, 641 natural village samples were selected. For those that cannot be investigated due to special reasons during the implementation process, the nearest natural village had been selected for replacement according to the sampling principle. The sampling of households did not adopt the conventional household registration roster method but adopted the “right-hand principle”. The first household in the village had been used as the starting point for interviews. After successful sampling, the next households had contacted at intervals of 3–5 households. Only 15–25 households were required to be sampled, that was, the minimum household sample in a village had 15 households, and the maximum was 25 households. In terms of data cleaning, we eliminated the missing values of the sample data, and 6,248 samples were retained in our analysis.

### 2.3. Variables and statistical description

The dependent variable is rural residents' self-rated health status. To assess self-rated health, we asked the respondents, “In general, would you say your health status is excellent, very good, good, fair or poor?”. The Answers from “poor” to “excellent” is assigned as 1–5 respectively. The literature shows that self-rated health status is an overall assessment that can reflect respondents' health status. It includes objective indicators such as past medical history and takes into account more subjective feelings such as disease severity and stability. It is a relatively comprehensive and proper health indicator that reflects an individual's overall health status (30).

The explanatory variable is the intermittent water supply. Corresponding to continuous water supply, intermittent water supply refers to a water supply mode that does not meet the requirements of continuously providing water to users 24/7. We assign the value of 1 to the households under intermittent water supply, and assign the value of 0 to the households under continuous water supply.

Referring to the relevant literature (13, 26, 31), we construct the control variables from three aspects: individual characteristics, household characteristics, and water habits. Individual characteristics include gender, age, education level, political status, marital status, and residence time. Household characteristics include the household size, monthly disposable income, and monthly expenditure of the household. In terms of residents' water habits, we choose domestic water sources, drinking habits, sanitary habits, and water quality awareness variables.

The descriptive statistics of the main variables are shown in Table 1. In general, the sample areas are dominated by continuous water supply, and only about 16% are in the intermittent water supply. From the point of self-rated health status, the respondents are in relatively good health with an average value of the sample is 3.85. From the perspective of age composition, the aging problem is still very serious in rural areas. The average age of the sample is 51.82 years old. The population over the age of 60 accounts for 30.23%, and the population over the age of 65 accounts for 19.78%. Meanwhile, the average number of years of education is 7.35 years, and the sample with an education level at or below junior high school accounts for 87.87%. Only 3.78% of rural residents have a college degree or above. These have shown the education level is still generally low in rural China. The average household size of the sample is 3.49. The average monthly disposable income of the household is about CNY 1940, and the average monthly household expenditure is CNY 1703. For household domestic water, 74% of households use running water as the main water source. At the same time, the respondent rural residents' water habit is good in sanitation, which is embodied in the mean values of variables in drinking water habits, water habits, and water quality cognition are all above 0.7. In addition, respondents spent an average of 11.05 months at home in the past year, and 88.22% of respondents (5,513 households) lived at home throughout the past year. So, the respondents in our study have a better understanding of water supply and other conditions in the household. The survey data is highly reliable and representative.

**Table 1:** Summary statistics.

**Variable**	**Description**	**Mean**	**Standard deviation**	**Min**.	**Max**.
**Dependent variable**					
Self-rated Health	1 = poor, 2 = fair, 3 = good 4 = very good, 5 = excellent	3.85	1.01	1	5
**Core explanatory variable**					
Intermittent Water Supply	1 = intermittent water supply; 0 = continuous water supply	0.16	0.37	0	1
**Mediating variable**					
Water Storage Behavior	1 = has a water storage habit, 0 = no water storage habit	0.13	0.34	0	1
**Individual variables**					
Gender	1 = male, 0= female	0.59	0.49	0	1
Age	Respondent's age in 2020 (years)	51.82	14.52	14	88
Education Level	Respondent's years of education (years)	7.35	3.09	0	22
Political Status	1 = party member, 0 = other	0.06	0.23	0	1
Marital Status	1 = married, 0 = other	0.82	0.38	0	1
Residence Time	Time of stay at home in the past year (months)	11.05	2.76	0	12
**Household variables**					
Household Size	The number of people who live together in the family (person)	3.49	1.88	1	15
Household Monthly Disposable Income	Monthly disposable household income (unit: CNY), logarithm	7.57	0.95	4.61	11.51
Household Monthly Expenditure	Monthly household consumption expenditure (unit: CNY), logarithm	7.44	0.80	2.30	9.90
**Water habits variables**					
Source for Domestic Water	1 = running water, 0 = other	0.74	0.44	0	1
Drinking Habits	How often drinking unboiled water: 0 = often, 0.5= sometimes, 1 = never	0.79	0.36	0	1
Handwashing Before Meals	Whether to wash hands before meals: 1 = yes, 0 = no	0.88	0.32	0	1
Handwashing After Toilet	Wash hands after toilet: 1 = yes, 0 = no	0.90	0.30	0	1
Water Quality Cognition	The impact of daily drinking water quality on health, 1 = no impact, 2 = little impact, 3 = great impact	2.56	0.69	1	3

### 2.4. Methods

To explore the impact of intermittent water supply on the self-rated health status of farmers and its mechanism, we first conduct an ordered probit (Oprobit) model to test the baseline effect. And we introduce an instrumental variable and establish the IV-Oprobit (Instrumental Variable-Ordered probit) model to discuss the endogeneity issues of our estimation. Then we replace the estimation model and the sample to test the robustness of our estimation. Finally, we conduct the two-stage least squares model (2SLS) and group regressions to test the mechanisms in our study.

#### 2.4.1. Baseline regression: The ordered probit model

Our main objective is to examine the impact of intermittent water supply on the self-rated health. Since the self-rated health status is the data of sorting selection, we conduct the ordered probit model for parameter estimation. The empirical model set is as follows:


(8) $$\begin{array}{l} {Health}_{i}=\;a_{0}+a_{1}Watersupply_{i}+a_{2}X_{i}+\lambda_{c}+\mu_{i} \end{array}$$

Where *Health*_*i*_ is the self-rated health status of the farmer *i*; *Watersupply*_*i*_ represents the water supply method of the village where the farmer *i* lives. *X*_*i*_ is a series of control variables, including the characteristics of individuals, households and the variables of water habits. Besides, we control the provincial fixed effects λ_*c*_; μ_*i*_ is a stochastic disturbance term. Assuming μ_*i*_ ~ N (0, 1), the ordered probit model can be expressed as:


$$\begin{array}{l} P\left(Health=1\;|\;x\right)=P\left(Health^{{}^{*}}\leq r_{0}\;|\;x\right)\\ \;=\varphi \left(r_{0}-a_{1}Watersupply_{i}s-a_{2}X_{i}\right) \end{array}$$

$$\begin{array}{l} P\left(Health=2\;|\;x\right)=P\left(r_{0}<Health^{{}^{*}}\leq r_{1}\;|\;x\right)\\ \;=\varphi \left(r_{1}-a_{1}Watersupply_{i}-a_{2}X_{i}\right)\\ \;-\varphi \left(r_{0}-a_{1}Watersupply_{i}-a_{2}X_{i}\right)... \end{array}$$

$$\begin{array}{l} P\left(Health=5\;|\;x\right)=P\left(Health^{{}^{*}}\leq r_{3}\;|\;x\right)\\ \;=1-\varphi \left(r_{3}-a_{1}Watersupply_{i}-a_{2}X_{i}\right) \end{array}$$

Among them, *r*_0_ < *r*_1_ < *r*_2_ < *r*_3_, is the parameter to be estimated; *Health*_*i*_ takes the value of 1, 2, 3, 4, and 5, which represents “poor”, “fair”, “good”, “very good” and “excellent” in health, respectively. By constructing the likelihood function model of the self-rated health status, we use the maximum likelihood estimation method to estimate the parameters of the model.

#### 2.4.2. Mechanism examination

Based on the theoretical analysis, referring to the suggestions of Jiang (32), we discuss the adverse effects of water storage behavior on self-rated health theoretically. In the empirical analysis, we focus more on the examination of the effect of intermittent water supply on residents' water storage behavior to test the potential mechanism. To better solve the endogeneity issues caused by the potential omitted variables, we establish the following estimations:


$$\begin{array}{l} Watersupply_{i}\text{\_}hat=\;a_{0}+a_{1}Population_{i}\\ \;+\;a_{2}X_{i}+\lambda_{c}+\varepsilon_{i} \end{array}$$

$$\begin{array}{l} Storage_{i}=\beta_{0}+\beta_{1}Watersupply_{i}\text{\_}hat+\beta_{2}X_{i}+\lambda_{c}+\mu_{i} \end{array}$$

Where the instrumental variable *Population*_*i*_ is the average respondent household size within a village. The selection and demonstration of the instrumental variable will be further elaborated in the empirical analysis below, Section Discussion. We substitute the predicted value of intermittent water supply estimated by the first-stage regression *Watersupply*_*i*__*hat* into the second-stage regression, in which β_1_ is the net effect of the water supply on rural residents' water storage behavior obtained by the 2SLS estimation. The settings of other variables are the same as the baseline regression.

In addition, we conduct the KHB method to decompose the total effect and identify the mechanisms (33). In our estimation, Oprobit is a nested non-linear probability model, uncontrolled and controlled coefficients can differ not only because of confounding but also a rescaling of the model. The difference in coefficients arises whenever the mediator variable has an independent effect on the dependent variable. To solve this problem, we refer to the KHB method proposed by Kohler, Karlson, and Holm to decompose the total effect into direct effect and indirect effect, which makes the results conform more precisely to the real coefficient difference and makes the examination of the mechanisms more intuitive (33, 34).

We conduct group regressions to discuss whether the water supply has influenced the self-rated health of rural residents with different sanitary water habits. First, we measure the level of individual hygiene water habits by calculating the average levels of drinking habits, hand washing before meals and after toileting habits. Then we take the mean value of sanitary water habits in the whole sample as a boundary, and classify those higher than the mean as the sample with better hygiene water habits group. Those below the mean are assigned to the sample group with poor sanitary water habits. Finally, we use the Oprobit model and the IV-Oprobit model for empirical testing and analysis. The setting of the model and other variables is the same as the baseline effect analysis.

## 3. Results

### 3.1. The baseline effects

We use Stata 16.0 for statistical analysis and column (1) in Table 2 below shows the baseline effects of intermittent water supply on the self-rated health status of rural residents. The results show that the intermittent water supply has a significant negative impact on the self-rated health status and the coefficient is statistically significant at 1%, which means that in the intermittent water supply areas, rural residents' self-rated health status is significantly worse compared with those in continuous water supply areas.

**Table 2:** Baseline effects of intermittent water supply on the self-rated health status.

**Variable**	**(1)**	**(2)**	**Marginal effects (IV-Oprobit)**				
	**Oprobit**	**IV-Oprobit**	**Poor**	**Fair**	**Good**	**Very good**	**Excellent**
Intermittent water supply	−0.162^***^	−1.873^***^	0.152	0.182^***^	0.204^***^	0.050	−0.588^***^
	(0.041)	(0.460)	(0.097)	(0.007)	(0.018)	(0.045)	(0.160)
Gender	0.116^***^	0.091^***^	−0.007^***^	−0.009^**^	−0.010^**^	−0.002^*^	0.029^***^
	(0.029)	(0.028)	(0.002)	(0.004)	(0.004)	(0.002)	(0.008)
Age	−0.020^***^	−0.016^***^	0.001^***^	0.002^**^	0.002^***^	0.000^**^	−0.005^***^
	(0.001)	(0.003)	(0.000)	(0.001)	(0.001)	(0.000)	(0.001)
Education level	0.027^***^	0.021^***^	−0.002^***^	−0.002^**^	−0.002^**^	−0.001^**^	0.006^***^
	(0.005)	(0.006)	(0.000)	(0.001)	(0.001)	(0.000)	(0.002)
Political status	0.156^**^	0.122^**^	−0.010^**^	−0.012^*^	−0.013^*^	−0.003	0.038^**^
	(0.063)	(0.054)	(0.005)	(0.007)	(0.007)	(0.002)	(0.019)
Marital status	−0.015	−0.014	0.001	0.001	0.001	0.000	−0.004
	(0.038)	(0.030)	(0.002)	(0.003)	(0.003)	(0.001)	(0.009)
Residence time	−0.012^**^	−0.009^**^	0.001^**^	0.001^*^	0.001^*^	0.000	−0.003^**^
	(0.005)	(0.004)	(0.000)	(0.001)	(0.001)	(0.000)	(0.001)
Household size	0.008	0.015^**^	−0.001	−0.001^**^	−0.002^**^	−0.000	0.005^**^
	(0.008)	(0.007)	(0.001)	(0.001)	(0.001)	(0.000)	(0.002)
Household monthly disposable income	0.180^***^	0.141^***^	−0.011^***^	−0.014^**^	−0.015^***^	−0.004^**^	0.044^***^
	(0.023)	(0.031)	(0.003)	(0.006)	(0.006)	(0.002)	(0.009)
Household monthly expenditure	−0.141^***^	−0.111^***^	0.009^***^	0.011^**^	0.012^***^	0.003^**^	−0.035^***^
	(0.027)	(0.028)	(0.003)	(0.005)	(0.005)	(0.002)	(0.008)
Source for domestic water	0.101^***^	0.098^***^	−0.008^**^	−0.010^**^	−0.011^**^	−0.003^*^	0.031^***^
	(0.035)	(0.029)	(0.003)	(0.004)	(0.004)	(0.002)	(0.009)
Drinking water habits	−0.121^***^	−0.093^**^	0.007^**^	0.009^*^	0.0010^**^	0.002^*^	−0.029^***^
	(0.042)	(0.036)	(0.003)	(0.005)	(0.005)	(0.001)	(0.011)
Handwashing before meals	0.175^***^	0.145^***^	−0.012^***^	−0.014^**^	−0.016^**^	−0.004^*^	0.045^***^
	(0.048)	(0.043)	(0.004)	(0.007)	(0.006)	(0.002)	(0.013)
Handwashing after toilet	0.037	0.034	−0.003	−0.003	−0.004	−0.001	0.011
	(0.051)	(0.039)	(0.003)	(0.004)	(0.004)	(0.001)	(0.012)
Water quality cognition	0.086^***^	0.066^***^	−0.005^***^	−0.006^**^	−0.007^**^	−0.002^*^	0.021^***^
	(0.023)	(0.021)	(0.002)	(0.003)	(0.003)	(0.001)	(0.006)
Control of regions	Controlled	Controlled	Controlled	Controlled	Controlled	Controlled	Controlled
Pseudo R^2^/Lnsig_2	0.100	−1.023^***^					
		(0.009)					
Lr Chi^2^ /Atanhrho_12	1,683.71	0.744^**^					
	(0.000)	(0.289)					
Log pseudolikelihood / Wald chi2	−7,548.94	4,858.05					
		(0.000)					
Number of obs.	6,248	6,248	6,248				

^*^p < 0.10, ^**^p < 0.05, ^***^p < 0.01, the robust standard errors are clustered in brackets. lnsig_2 is the significance test value of the second stage estimation, and atanhrho_12 is the correlation test of the error terms of the first and second stage estimation.

In terms of individual characteristic variables, such as gender, age, education level, and political status all significant impact on the self-rated health status of rural residents. Among them, the older the rural resident is, the self-rated health status is getting poorer, of which the coefficient is statistically significant at 1%. As age increases, the probability of “excellent” health status reduces by 0.5%, and the probability of “fair” status increases by 0.2%. It is also in line with the prediction based on the Grossman model. The increase in age increases the depreciation rate, the equilibrium point moves up, and then shows a corresponding decline in demand for health. At the same time, with the increase of age, body function gradually declines and the probability of suffering from various diseases also increases. In addition, gender is also a significant influence factor on self-rated health status. Compared with females, male residents' self-rated health status is better in varying degrees, and the coefficient is also statistically significant at 1%. Moreover, the education level and political status have a significant positive effect on the individual's self-rated health status at the 1 and 5% confidence levels, respectively, which is also in line with the analysis based on the Grossman model. Education and health as two complementary human capital, the improvement of education level can improve the efficiency of health production, reduce the shadow price of health, and make the health benefit curve move upwards, resulting in the level of demand for health in new equilibrium increases. Similarly, for party members, the subtle influence of the environment, such as households and various regulations, makes them more likely to abandon unhealthy living habits, thereby their self-rated health status is generally better than non-party members.

From the perspective of household characteristics, household size has a significant positive impact on the self-rated health status of individuals. With the increase in the population size of households, the health status of residents has also been improved to varying degrees and the coefficient is statistically significant at 5%. In addition, the monthly disposable income of the household promotes the improvement of the self-rated health status] at the 1% confidence level. Especially, when the household monthly disposable income increases by 1%, the probability of the individual's self-rated health status of being “excellent” will increase by 4.4%. Income affects health demand through the health shadow price in the Grossman model. While the impact of monthly expenditure is the opposite. The probability of being “excellent” reduces by 3.5% with the household monthly expenditure increasing by 1%. The explanation from our study is that expenditure is an external economic manifestation of lifestyles, the rural households with higher expenditure tend to have non-thrifty lifestyles, of which the disadvantages to health outweigh the advantages.

As for residents' sanitary water behavior, the water source of households significantly promotes the improvement of an individual's self-rated health status at the 1% confidence level. The resident who uses running water as the main water source has a lower probability of being “fair” health, which significantly drops by 1%, while the odds of self-rated “excellent” would increase by 3.1%. This conclusion is also consistent with existing studies (13, 26), compared with other sources, running water is safer. It can not only reduce the risk of individuals suffering from infectious diseases, especially water-borne infectious diseases, to safeguard the physical health of rural residents but is also beneficial to the individual's mental health (35). The residents' cognition of water quality also significantly affects their self-rated health status at the 1% confidence level. With the cognition of water quality increasing by 1 unit, the probability of self-rated health being “excellent” would significantly increase by 2.1%. In other words, the cognition of water quality may affect residents' sanitary water behavior to a certain extent and thus affect their self-rated health status.

#### 3.1.1. Endogeneity issues

This study has controlled the relevant influencing factors as much as possible but still cannot avoid the endogeneity issues caused by omitted variables, which could result in estimate bias. Because the factors that affect personal health status are too complex, or some of them are too hard to observe. Therefore, drawing on the practice of Lu (36), we introduce an instrumental variable (IV-Oprobit model) to deal with the endogeneity issues.

As a shock from outside the model, instrumental variables should be highly correlated with endogenous explanatory variables and not correlated with the stochastic disturbance term. At this point, we choose “the average respondent household size within a village” as the instrumental variable in our estimation. Studies have shown that the rapid growth of the economy, population, and water demand is an important reason for the shortage of water resources, so intermittent water supply is introduced to limit the water consumption by residents (2, 3). Therefore, it can be considered that villages with larger populations face greater water demand and are more likely to adopt intermittent water supply to limit and regulate water consumption. Besides, according to the randomness of sampling, this paper believes that the average respondent household size within a village can represent the population size of the interviewed village to a certain extent. So, this variable satisfies the assumption of correlation regards the instrumental variable. At the same time, the average respondent household size within a village belongs to a variable at the village level, which will not be directly related to the health status of the individuals. In a word, it conforms to the assumptions of correlation and exogeneity and can be treated as an instrumental variable in our estimation.

Column (2) in Table 2 shows the results of the re-examination of the intermittent water supply's impact on health status using the IV-Oprobit model. The lnsig _2 value of the estimation is −1.023, and it is statistically significant at 1%, indicating that the second-stage estimation of the model is significant. Besides, our estimation has passed the atanhrho _12 test, indicating that our baseline regression results are biased, and the results estimated by the cmp method are better than those estimated by the Oprobit model, that is, the introduction of an instrumental variable in the Oprobit model is effective. From the estimation results, the intermittent water supply still significantly has a negative impact on rural residents' health. Meanwhile, the absolute value of the estimated coefficient of intermittent water supply is larger than the estimated coefficient in the baseline regression, indicating that potential endogeneity issues caused an underestimation of the adverse effects of intermittent water supply on individual health. Judging from the marginal effects reported in Table 1, the effect of intermittent water supply on the health status of rural residents is mainly reflected in the impact on “fair”, “good” and “excellent” status. Specifically, compared with rural residents under continuous water supply, the probability of “fair” will significantly increase by 18.2%, and the probability of “excellent” status will significantly reduce by 58.8%, all of which further verify Hypothesis 1. In addition, each control variable's influence on individual health status is similar to the baseline regression results reported in column (1), and the specific analysis will not be repeated here.

#### 3.1.2. Robustness test

To ensure the reliability of our results, this paper uses the method of replacing the estimation model and the sample to test the robustness of the baseline effect. Firstly, based on the previous analysis, this paper refers to the practice of Fang and Lu (37), dichotomizing the ordered variable of self-rated health status into binary variables. Specifically, we clarify the rural residents whose self-rated health status is “poor” or “fair” as a “less healthy” sample group (coded 0); the residents who are in “good”, “very good” or “excellent” status have been classified as a “healthier” group (coded 1). Therefore, this paper further establishes the probit model and the IV-probit model for the robustness test. The results are shown below in Table 3. In this estimation, since the number of endogenous explanatory variables is equal to the number of instrumental variables, the over-identification test is not required (38). The F-statistic value in the first stage regression is 42.80, which is much higher than the empirical value of 10. It can be considered that the problem of weak instrumental variables is not obvious (39). The regression results are shown in columns (5) and (6). The intermittent water supply still significantly reduces the self-rated health status of rural residents at the 1% significance level. After the introduction of instrumental variables in the IVprobit model, the marginal effect of the intermittent water supply on health is larger, indicating that the endogenous issues will also underestimate the impact of water supply time constraints on health. At the same time, the regression results of other control variables are consistent with the baseline estimation, indicating that the regression results are robust.

**Table 3:** Robustness test results.

**Dependent variable: Self-rated health**	**Full sample**				**Subsample**	
	**(3)**	**(4)**	**(5)**	**(6)**	**(7)**	**(8)**
	**Oprobit**	**IV-Oprobit**	**probit**	**IVprobit**	**Oprobit**	**IV-Oprobit**
Intermittent water supply	−0.162^***^	−1.873^***^	−0.028^***^	−0.182^**^	−0.141^***^	−1.826^***^
	(0.041)	(0.460)	(0.009)	(0.071)	(0.052)	(0.412)
Control variable	Controlled	Controlled	Controlled	Controlled	Controlled	Controlled
Control of regions	Controlled	Controlled	Controlled	Controlled	Controlled	Controlled
Pseudo R ^2^ / Lnsig_2	0.100	−1.023^***^	0.164	0.130	0.092	−1.004^***^
LR Chi ^2^ /Atanhrho_12	1,683.71	0.744^**^	611.83	494.51	748.99	0.749^***^
Log pseudolikelihood/Wald chi2	−7,548.94	4,858.09	−1,712.11	−1,781.17	−4,158.28	2,958.66
Number of obs.	6,248	6,249	6,248	6,248	3,664	3,664

^*^p < 0.10, ^**^p < 0.05, ^***^p < 0.01, the robust standard errors are clustered in brackets. The reported results of models (5) and (6) are the marginal effects; lnsig_2 is the significance test value of the second stage estimation; atanhrho_12 is the correlation test of the error terms of the first and the second stage estimation; results for control variables are not reported, those results are available upon request.

In addition, considering the impact of natural aging on health, we refer to Zhao (26) to restrict our sample between the ages of 18 and 55. The settings of variables and methods are the same as those in the baseline regression analysis. Columns (7) and (8) show the estimation results using the Oprobit and IV-Oprobit models, respectively. The intermittent water supply still has a significant negative effect on the health status of rural residents at the 1% confidence level. Meanwhile, the estimation results of other variables are consistent with the baseline estimation, which also shows that the estimation results of our study are robust.

### 3.2. Mechanism examinations

#### 3.2.1. Water storage behavior mechanism

When testing whether the water storage behavior is the mechanism that intermittent water supply affects the health status of rural residents, there will also avoidably be omitted variables using OLS estimation because the water storage behavior may be affected by many factors such as the personal internal characteristics and the growth environment, which will result in estimate bias. Therefore, this paper continues to follow the previous empirical strategy, using the “average respondent household size within a village” as the instrumental variable to solve the endogeneity issues. According to the above discussions, the average respondent household size within a village can represent the population size of the interviewed village to a certain extent. The larger the population size of the village will face the greater the water demand. The intermittent water supply has been used to limit and regulate residential water use. So, it accords with the correlation assumption of an instrumental variable. At the same time, as a predetermined fact at the village level, the population size of the village does not have a direct impact on the water storage behavior of the respondent individuals, which also satisfies the exogenous assumption. So, the construction of the instrumental variable is effective.

Model (A) in Table 4 reports the OLS estimation results of equation (1), and model (B) reports the 2SLS estimation results. After dealing with endogenous issues, the results show that the intermittent water supply still significantly promotes rural residents' water storage behavior. But the net effect of intermittent water supply on residents' water storage behavior estimated by 2SLS has decreased. In intermittent water supply areas, compared with continuous water supply, the probability of rural residents' water storage behavior has increased by 26.6%. In the OLS estimation, the impact is as high as 48.6%, which means the potential endogenous issues have overestimated the impact of intermittent water supply, and our empirical strategy of introducing an instrumental variable is effective.

**Table 4:** Examination of water storage behavior mechanism.

	**Model (A)**	**Model (B)**	
	**Water storage behavior**	**Intermittent water supply**	**Water storage behavior**
Intermittent water supply	0.486^***^	-	0.266^***^
	(0.015)	-	(0.069)
Average respondent household size within a village	-	0.054^***^	-
	-	(0.005)	-
Control variables	Controlled	Controlled	Controlled
Control of regions	Controlled	Controlled	Controlled
The first stage regression F-value	-	46.31	-
Number of obs.	6,248	6,248	6,248
R^2^	0.362	0.100	0.272

^*^p < 0.10, ^**^p < 0.05, ^***^p < 0.01, the robust standard errors of the coefficients are in parentheses, and the data in the table are the results after rounding; Results for control variables are not reported, those results are available upon request.

Besides, previous studies have demonstrated that water storage behavior has adverse effects on the individual health of residents from the aspects of water quality and water storage sanitation (3, 18, 20). Therefore, combined with the previous demonstrations in this paper, it can be concluded that water storage behavior is an important mechanism for the influence of water supply on residents' health, and Hypothesis 2 is verified. Further, Table 5 reports the decomposition results of the main and indirect effects using the KHB method. The results show that the coefficient of the intermittent water supply's total effect on the health status of rural residents is 0.059, which is significant at the 1% confidence level. The coefficient of the direct effect is 0.031 and statistically significant at 10%. The coefficient of the indirect effect is 0.028 and statistically significant at 5%, accounting for 47.46% of the total effect. The decomposition results further illustrate that water storage behavior is an important mechanism for the influence of intermittent water supply on rural residents' self-rated health status.

**Table 5:** Total effect decomposition based on KHB method.

	**Coefficient**	**Standard error**	***P* > |*z*|**	**[95% confidence interval]**		**Share of the total effect**
Total Effect	−0.059^***^	0.015	0.000	−0.088	−0.031	100.00%
Direct Effect	−0.031^*^	0.017	0.070	−0.065	0.002	52.54%
Indirect Effect	−0.028^**^	0.012	0.021	−0.053	−0.004	47.46%

^*^p < 0.10, ^**^p < 0.05, ^***^p < 0.01.

#### 3.2.2. Sanitary water habit mechanism

Table 6 below reports the results of the test on the sanitary water habit mechanism. Model (A) uses the Oprobit model, and Model (B) uses the IV-Oprobit model for estimation. Among them, in the Oprobit model, the intermittent water supply has a significant impact on the health status of the respondents with both poor and good water sanitary habits, and the coefficients are statistically significant at 1 and 10%, respectively. The influence on the health of the rural residents with poor sanitary water habits is more significant, and the estimation coefficient is also larger. Since the coefficients of the two groups are significant at different confidence levels, which makes it unreliable to compare the two coefficients directly. We refer to the practice of Lian (40), using the “bootstrap” method to test the significance of the difference in coefficients between groups. We find that the empirical *p*-value of the difference between groups is 0.50 after 1,000 self-samplings, and it is statistically significant at 5%, which further proves the statistical significance of the different coefficients between the groups. However, after solving the endogeneity issues, in the sample group with poor sanitary water habits, the intermittent water supply still has a significant impact on the health status of residents, but the effect is no longer significant in the sample group with better sanitary water habits. Overall, sanitary water habit is also an important mechanism for the influence of intermittent water supply on the health status of rural residents. When residents with poor sanitary water habits, the adverse effects of intermittent water supply on residents' health are getting more significant.

**Table 6:** Examination of sanitary water habit mechanism.

**Dependent Variable: Self-Rated Health**	**(A): Oprobit**		**(B): IV-Oprobit**	
	**Poor sanitary water habits**	**Better sanitary Water habits**	**Poor sanitary water habits**	**Better sanitary Water habits**
Intermittent water supply	−0.218^***^	−0.098^*^	−1.832^***^	−0.655
	(0.059)	(0.057)	(0.364)	(1.082)
Control variables	Controlled	Controlled	Controlled	Controlled
Control of regions	Controlled	Controlled	Controlled	Controlled
Pseudo R^2^/Lnsig_2	0.062	0.121	−0.927^***^	−1.051^***^
LR Chi^2^ /Atanhrho_12	436.41	1,172.37	0.808^***^	0.199
Log pseudolikelihood/Wald chi2	−3,134.35	−4,403.11	1,216.57	1,228.54
Empirical *p-*value	0.050^**^		-	
Number of obs.	2,469	3,779	2,469	3,779

^*^p < 0.10, ^**^p < 0.05, ^***^p < 0.01, the robust standard errors are clustered in brackets; lnsig_2 is the significance test value of the second stage estimation; atanhrho_12 is the correlation test of the error terms of the first and second stage estimation; “empirical p-value” is used to test the significance of coefficient difference of intermittent water supply between groups, which is obtained by 1000 times of self-sampling (bootstrap); results for control variables are not reported, those results are available upon request.

## 4. Discussion

The purpose of health economics research is not only to maximize the benefits of medical and health resources, but also to improve the human capital of groups, especially to realize the transformation of the health status of different groups from heterogeneity to homogeneity (41). Literature has shown that changes in the physical environment of health investment will have different effects on the health of different people. For example, the rural toilet renovation project in China has a more significant positive effect on female's self-rated health (42); while the impact on rural residents with higher health levels is not significant (43). Residential energy resource replacement has higher health returns for women, the elderly, and illiterate groups (37). Similarly, the intermittent water supply also affects individuals' health through the physical environment of residents' health investments. Further exploration of the impact on the health status of different groups of residents will provide a meaningful reference for the formulation of policies and measures in the future. Therefore, we divide the sample into different subgroups by gender and education level to further examine the impact of intermittent water supply on individual health status.

The respondents are firstly grouped by gender, and the impact of intermittent water supply on health status is estimated in both male and female groups. Table 7 shows the estimation results that intermittent water supply has a significant negative impact on the health status of both female and male residents. The coefficients are statistically significant at the 1 and 10% confidence levels, respectively. Among them, the coefficient of the intermittent water supply in the female group is larger than that in the male group. The difference between the two coefficients is statistically significant, with an empirical *p-*value of 0.03. On this basis, we report the marginal effects of the intermittent water supply on the health status of different groups in Table 7. We find that in the female group, compared with continuous water supply, the probability of residents becoming “fair” under intermittent water supply has significantly increased by 22.3%; while the probability of being “excellent” has be significantly reduced by 87.3%, compared with 14.6 and 39.2% in the male group, respectively. The results are also consistent to some peer studies (42, 43). The greater impact of water supply on the health of the female group may be related to the role that most women are responsible for daily issues in the household, they are more often to deal with domestic water situations and are more vulnerable.

**Table 7:** Effects of water supply on self-rated health status in different subgroups.

	**Dependent variable: self-rated health status (IV-Oprobit)**			
	**Female**	**Male**	**Lower education level**	**Higher education level**
Intermittent water supply	−2.667^***^	−1.279^*^	−1.968^***^	−1.842^*^
	(0.367)	(0.656)	(0.474)	(0.978)
Control variables	Controlled	Controlled	Controlled	Controlled
Control of regions	Controlled	Controlled	Controlled	Controlled
Lnsig_2	−1.068^***^	−1.051^***^	−1.008^***^	−1.048^***^
LR Chi^2^ /Atanhrho_12	1.516^**^	0.429	0.872^**^	0.626
Log pseudolikelihood/Wald chi2	5,742.47	1,544.84	3,266.35	1,622.00
Empirical *p-*value	0.003^***^		0.042^**^	
Number of obs.	2,564	3,684	3,650	2,598

^*^p < 0.10, ^**^p < 0.05, ^***^p < 0.01, the robust standard errors are clustered in brackets; lnsig_2 is the significance test value of the second stage estimation; atanhrho_12 is the correlation test of the error terms of the first and the second stage estimation; “empirical p-value” is used to test the significance of coefficient difference of intermittent water supply between groups, which is obtained by 1000 times of self-sampling (bootstrap); results for control variables are not reported, those results are available upon request.

Meanwhile, we divide the respondents into two subgroups according to their education level: a higher education level group with a degree of junior high school or above. A lower education level group with a degree in primary school or below. Table 7 shows the estimation results that the intermittent water supply has a significant negative impact on the health status of different education level groups, and the difference between the coefficients in the two groups is statistically significant. We report the marginal effect of water supply on the health status of different education level groups in Table 8. Different from the analysis of gender subgroups, the difference in the impact on the health status of different educational groups is mainly reflected in the change of “unhealthy” status. Compared with the areas with continuous water supply, the probability of “very unhealthy” in the lower education level group under intermittent water supply conditions has significantly increased by 20.4%. The probability of “not very healthy” has significantly increased by 20.9%. However, this effect has only been reflected in the “less healthy” status in the higher education level group with a magnitude of 13.0%. We could also find similar results in the study of residential energy resource replacement, which also worked on the change of physical conditions in health investment (37).

**Table 8:** Marginal effects of water supply on self-rated health in different groups.

	**Marginal effects (IV-Oprobit)**					
**A: Group by gender**	**Fair**		**Very good**		**Excellent**	
	**Female**	**Male**	**Female**	**Male**	**Female**	**Male**
Intermittent water supply	0.223^***^	0.146^***^	0.096^***^	−0.392^***^	−0.873^***^	−0.392^***^
	(0.028)	(0.054)	(0.022)	(0.207)	(0.116)	(0.207)
Control variables	Controlled	Controlled	Controlled	Controlled	Controlled	Controlled
Control of regions	Controlled	Controlled	Controlled	Controlled	Controlled	Controlled
Number of Obs.	2,564	3,684	2,564	3,684	2,564	3,684
**B: Group by education levels**	**Poor**		**Fair**		**Excellent**	
	**Lower education level**	**Higher education level**	**Lower education level**	**Higher education level**	**Lower education level**	**Higher education level**
Intermittent water supply	0.204^*^	0.108	0.209^***^	0.130^***^	−0.618^***^	−0.589^*^
	(0.121)	(0.163)	(0.011)	(0.014)	(0.173)	(0.322)
Control variables	Controlled	Controlled	Controlled	Controlled	Controlled	Controlled
Control of regions	Controlled	Controlled	Controlled	Controlled	Controlled	Controlled
Number of obs.	3,650	2,598	3,650	2,598	3,650	2,598

^*^p < 0.10, ^**^p < 0.05, ^***^p < 0.01, the robust standard errors are clustered in brackets; based on research needs and space limitations, only the marginal effects of intermittent water supply on specific health status are reported here; the results for control variables are not reported, those results are available upon request.

In general, based on the results in Tables 7, 8, this paper can draw the following conclusions: (1) The self-rated health status of female and lower education level groups is more vulnerable to being affected by the intermittent water supply. (2) The influence of intermittent water supply on the health status of groups with different gender has been reflected in the status of “fair” and “excellent”. While the influence on the groups with different education levels has mainly reflected in the health status of “poor” and “fair”, which belong to relatively unhealthy conditions.

## 5. Conclusion

With rapid economic and social development, the demand for water is surging, and the shortage of water resources is also increasing. The application of intermittent water supply systems has become more common in rural China and other developing countries, and the accompanying health risks require more attention. Based on the Grossman model, this paper empirically tested the impact of intermittent water supply on the health status of rural residents, using the data of the “China Karst Rural Economic Survey (CKRS)”. The study found: (1) Intermittent water supply has adverse impacts on the health status of rural residents: compared with rural residents under continuous water supply, the probability of “unhealthy” residents under intermittent water supply has significantly increased by 18.2%; while the probability of “very healthy” conditions would be significantly decreased by 58.8%. (2) Water storage behavior and sanitary water habit are important mechanisms for intermittent water supply to affect residents' health: intermittent water supply increases health risks by promoting rural residents' water storage behavior, which exposes the individuals to an environment with higher hygiene risks. In addition, the adverse effects of intermittent water supply on health would be more significant when residents have developed poor sanitary water habits. (3) There are certain differences in the impact of intermittent water supply on the health of different groups. The impact on the health status of female and lower education level groups is much larger. Specific groups in the water supply policy need more consideration. Based on the findings, this paper draws the following policy implications.

Firstly, rationally planning the water supply method and optimizing the water management system. Poor later-stage management is the main reason for the lack of water supply in China's karst areas. Some areas have just completed the installation of water pipes (until the date of our survey), but have not been officially put into practice, or the work is still unstable. So, there is an unstable water supply time in these areas. Relevant departments should rationally plan water supply modes, especially optimize the water management system, to ensure the normal use of running water in karst areas. Besides, part of the reason for the intermittent water supply in China's karst rural areas is the “engineering water shortage” problem, which has not been fully resolved for years. Only by improving the construction of water conservancy projects, fully developing shallow water sources, and reducing the loss of surface water, can we fundamentally solve the problem of water shortage and release the current dilemma of insufficient water supply. Finally, we should attach great importance to water quality, including strengthening the daily maintenance of the water supply pipe network, and strengthening the supervision of water quality tests; we should also choose different disinfection methods according to the characteristics of water quality in various places to minimize the microbial pollution, ensure drinking water safety according to local conditions, and reduce the hygiene risks caused insufficient water supply.

Secondly, strengthening water health education for rural residents and standardizing their sanitary water behavior. We should enhance residents' awareness of water hygiene, and guide residents to develop sanitary water habits, including drinking running water after boiling, washing hands before meals and after toileting, etc. Especially, in intermittent water supply areas, we should guide residents to properly release water before using it when the water supply is restored. At the same time, we should attach great importance to regulating residents' water storage behavior, and guide them not to use stored water as a direct source of drinking water to reduce the potential hygiene risks.

Thirdly, the pertinence of policy implementations should be enhanced, and specific measures should be tilted toward specific groups. As it stands today in rural China, the number of left-behind elderly and left-behind females and children is increasing, where the education level is generally low and the concept of health, knowledge of water sanitation are relatively deficient. The health status of females and groups with lower education levels is more likely to be affected by the change in the water supply system. Therefore, the promotion of safeguard measures should tilt toward specific groups in the intermittent water supply areas, making full use of various publicity channels such as broadcasting and online media to strengthen health publicity and sanitary water education in daily life. At the same time, for groups with lower educational levels, we should simplify the publicity and education methods to make them easy to understand. Measures such as posters and door-to-door publicity can be used to effectively enhance the pertinence of policy implementation, promote rural residents to form health concepts and good sanitary water habits, and increase the demand for more health services.

## Data availability statement

The raw data supporting the conclusions of this article will be made available by the authors, without undue reservation.

## Author contributions

GW supervised this study and provided the funding for this study. FY contributed in data analysis and provided step-by-step guidance to main author. JC is the main writer of this manuscript. NR reviewed this article, made improvements, and dealed all the requirements for final submission. All authors contributed to the article and approved the submitted version.
